# Uptake and Metabolism of the Novel Peptide Angiotensin-(1-12) by Neonatal Cardiac Myocytes

**DOI:** 10.1371/journal.pone.0015759

**Published:** 2011-01-10

**Authors:** Sarfaraz Ahmad, Jasmina Varagic, Brian M. Westwood, Mark C. Chappell, Carlos M. Ferrario

**Affiliations:** 1 Hypertension and Vascular Research Center, Wake Forest University School of Medicine, Winston-Salem, North Carolina, United States of America; 2 Division of Surgical Sciences, Wake Forest University School of Medicine, Winston-Salem, North Carolina, United States of America; 3 Department of Physiology/Pharmacology, Wake Forest University School of Medicine, Winston-Salem, North Carolina, United States of America; 4 Department of Internal Medicine/Nephrology, Wake Forest University School of Medicine, Winston-Salem, North Carolina, United States of America; University of Colorado Denver, United States of America

## Abstract

**Background:**

Angiotensin-(1–12) [Ang-(1–12)] functions as an endogenous substrate for the productions of Ang II and Ang-(1–7) by a non-renin dependent mechanism. This study evaluated whether Ang-(1–12) is incorporated by neonatal cardiac myocytes and the enzymatic pathways of ^125^I-Ang-(1–12) metabolism in the cardiac myocyte medium from WKY and SHR rats.

**Methodology/Principal Findings:**

The degradation of ^125^I-Ang-(1–12) (1 nmol/L) in the cultured medium of these cardiac myocytes was evaluated in the presence and absence of inhibitors for angiotensin converting enzymes 1 and 2, neprilysin and chymase. In both strains uptake of ^125^I-Ang-(1–12) by myocytes occurred in a time-dependent fashion. Uptake of intact Ang-(1–12) was significantly greater in cardiac myocytes of SHR as compared to WKY. In the absence of renin angiotensin system (RAS) enzymes inhibitors the hydrolysis of labeled Ang-(1–12) and the subsequent generation of smaller Ang peptides from Ang-(1–12) was significantly greater in SHR compared to WKY controls. ^125^I-Ang-(1–12) degradation into smaller Ang peptides fragments was significantly inhibited (90% in WKY and 71% in SHR) in the presence of all RAS enzymes inhibitors. Further analysis of peptide fractions generated through the incubation of Ang-(1–12) in the myocyte medium demonstrated a predominant hydrolytic effect of angiotensin converting enzyme and neprilysin in WKY and an additional role for chymase in SHR.

**Conclusions/Significance:**

These studies demonstrate that neonatal myocytes sequester angiotensin-(1–12) and revealed the enzymes involved in the conversion of the dodecapeptide substrate to biologically active angiotensin peptides.

## Introduction

Advances in the biochemical physiology of tissue renin angiotensin systems (RAS) now document the existence of alternate pathways for the generation and metabolism of angiotensin peptides. Adding to this knowledge are new findings showing that additional alternate mechanisms exist for the formation of angiotensin peptides upstream from angiotensin I (Ang I). Nagata et al. [1] identified an extended form of Ang I present in multiple organs of Wistar rats. The peptide named “proangiotensin-(1–12)” contained the sequence of Ang I plus -Leu^11^-Tyr^12^ at the C-terminus. In keeping with the nomenclature approved by the Council for High Blood Pressure Research [2] we refer to proangiotensin-(1–12) as angiotensin-(1–12) [Ang-(1–12)]. In their studies Nagata et al. [1] reported the capacity of the docadecapeptide to serve as a functional substrate for the production of angiotensin II (Ang II). The delivery of synthetic Ang-(1–12) either systemically or following application to isolated rat aorta induced pressor or vasoconstrictor responses that were eliminated by prior administration of the angiotensin converting enzyme (ACE) inhibitor captopril or the Ang II AT_1_ receptor antagonist, candesartan. Intrigued by the potential significance of Ang-(1–12) as an endogenous alternate precursor for the formation of Ang I, a series of studies from our laboratory documented the expression of Ang-(1–12) in the heart and kidney of Wistar Kyoto (WKY) and spontaneously hypertensive (SHR) rats [3] and showed in the isolated heart of three normotensive (Sprague Dawley, Lewis, and WKY) and two hypertensive (congenic mRen2.Lewis and SHR) rats that cardiac formation of Ang I, Ang II, and angiotensin-(1–7) [Ang-(1–7)] occurred through cleavage of Ang-(1–12) via a non-renin pathway. [4] Additional evidence for a renin independent formation of Ang II from Ang-(1–12) was demonstrated in anephric rats as the fall in circulating Ang I and Ang II was associated with increased cardiac content of Ang-(1–12) and Ang II 48 h post-bilateral nephrectomy. [5]

The scientific questions addressed in these studies are: a)- is Ang-(1–12) contained within and incorporated by neonatal cardiac myocytes?; b)- what are the routes of Ang-(1–12) metabolism in the medium of serum deprived cardiac myocytes?; and c)- is there a differential incorporation and routes of metabolism in normotensive WKY and SHR rats?

## Results

### Myocyte Uptake of ^125^I-Ang-(1–12)

**Figure 1:**
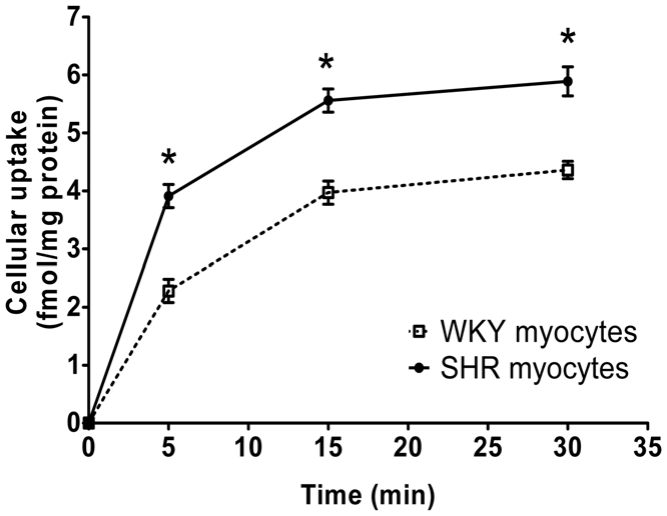
Uptake of Ang-(1–12) by neonatal cardiac myocytes. Time dependent increase in cellular uptake of ^125^I-Ang-(1–12) in cultured neonatal SHR cardiac myocytes (solid line) as compared to WKY (dotted line) in the presence of all RAS enzymes inhibitors. Cellular internalization of ^125^I-Ang-(1–12) is expressed as fmol^−1^ x mg^−1^ protein. Each value represents the mean ± SE of three or more independent assays. In these experiments, WKY and SHR myocytes were treated with all inhibitor cocktail containing lisinopril (ACE inhibitor), SCH39370 (NEP inhibitor), MLN-4760 (ACE2 inhibitor), and chymostatin (chymase inhibitor), peptidase inhibitors (amastatin, bestatin & benzyl succinate), and PCMB each added at a dose of 10 µM. *Significantly different (*P*<0.001) from the mean of the corresponding time point of WKY.

Total cellular uptake of 1 nM ^125^I-Ang-(1–12) by WKY and SHR myocytes were investigated in the presence of all RAS and peptidases inhibitors (amastatin, bestatin, benzyl succinate and PCMB). Figure 1 show that ^125^I-Ang-(1–12) was incorporated by cultured myocytes in a time-dependent fashion in both WKY and SHR. Importantly, the data shown in Figure 1 reveals that the rate of ^125^I-Ang-(1–12) uptake at all time points was significantly higher in SHR as compared to WKY myocytes. Based on cellular counts of ^125^I-Ang-(1–12) from the myocytes' lysate, peak cellular uptake of ^125^I-Ang-(1–12) averaged 0.78±0.08 fmol·mg protein^−1^·min^−1^ in SHR myocytes and 58% less in WKY myocytes (0.46±0.07 fmol·mg protein^−1^·min^−1^; p<0.001). Cellular counts of ^125^I-Ang-(1–12) in both WKY and SHR cell lysate were significantly decreased (around 50%) by a 10^3^-fold excess amount of unlabeled Ang-(1–12). These findings confirm and extend our initial report for an increased content of Ang-(1–12) in the heart of SHR through independent assessment of peptide content by both immunohistochemistry and radioimmunoassay (RIA) measurements. [3]

**Figure 2:**
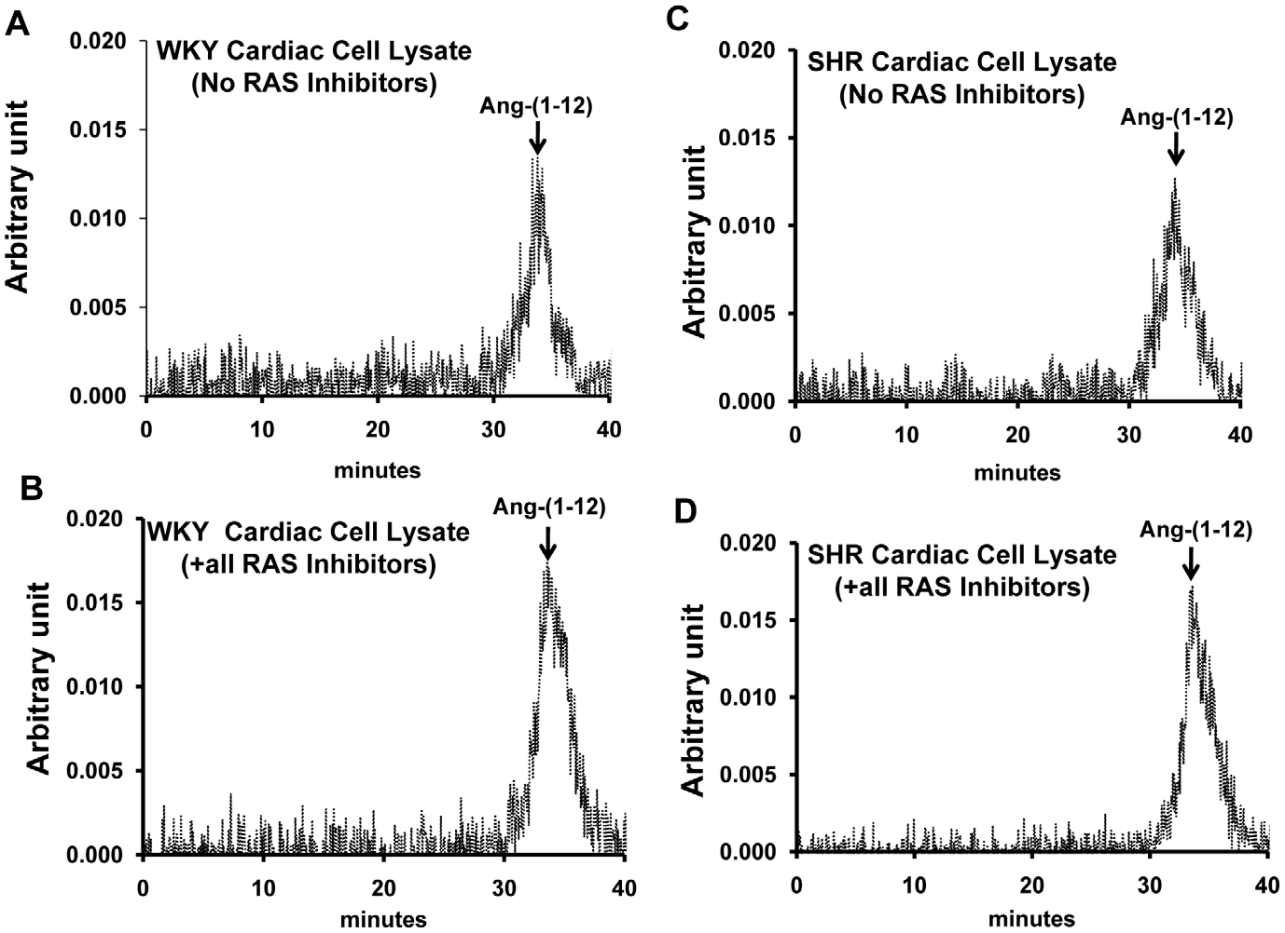
Effect of inhibitors on Ang-(1–12) metabolism in cardiac myocytes. Characterization by high pressure liquid chromatography of cellular ^125^I-Ang-(1–12) products internalized by 24-h serum deprived cultured neonatal WKY (A and B) and SHR (C and D) cardiac myocytes in the presence of all inhibitors cocktail (containing lisinopril, SCH39370, MLN-4760, chymostatin, amastatin, bestatin, benzyl succinate and PCMB) and in the absence of RAS inhibitors (containing only amastatin, bestatin, benzyl succinate & PCMB) each added at a dose of 10 µM. Before adding the ^125^I-Ang-(1–12), the cultured cardiac myocytes were pre-incubated with inhibitors for 15 min at 37°C. The arrow indicates the peak area of ^125^I-Ang-(1–12).

The cell lysate (cytosolic fraction) from cardiac myocytes samples was also analyzed by HPLC/radioisotope detection to ensure that intact ^125^I-Ang-(1–12) is internalized by these cells. Figure 2 shows that the majority of the cell-associated radioactivity eluted at a retention time identical to ^125^I-Ang-(1–12) in cardiac myocytes lysates of both strains in the absence (*No RAS inhibitor group*) and in the presence of all RAS inhibitors (ACE, NEP, ACE2, and chymase). These data confirmed that intact ^125^I-Ang-(1–12) is incorporated into the cardiac myocytes.

Total cellular internalized content of ^125^I-Ang-(1–12) in the presence or absence of all RAS enzyme inhibitors was calculated by analyzing the ^125^I-Ang-(1–12) peak of WKY and SHR myocytes. In the presence of all RAS inhibitors, the cellular content of ^125^I-Ang-(1–12) was 3.6±0.3% in WKY and 5.2±0.5% in SHR of the total loaded content of radiolabeled Ang-(1–12). However, in the absence of RAS inhibitors (*No RAS inhibitor group*), the cellular content of ^125^I-Ang-(1–12) was significantly lower (2.1±0.4% in WKY and 2.5±0.3% in SHR; *p*<0.05) as compared to the total ^125^I-Ang-(1–12) added to the reaction mixture.

### ^125^I-Ang-(1–12) Metabolism

**Figure 3:**
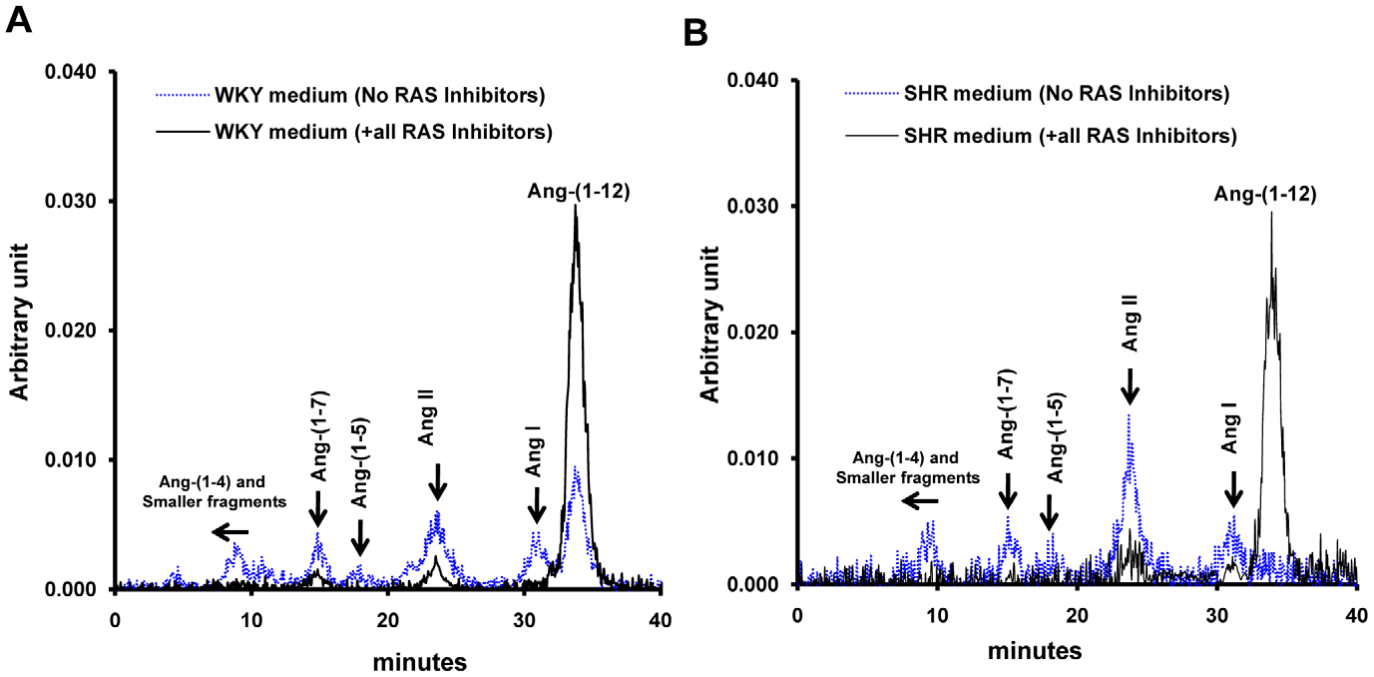
Patterns of Ang-(1–12) metabolism in the medium collected from cardiac myocytes. HPLC characterization of ^125^I-Ang products generated in the medium of cultured neonatal WKY (A) and SHR (B) cardiac myocytes exposed to ^125^I-Ang-(1–12) at 37°C for 60 min in the presence (solid line) and absence (dotted line) of the RAS inhibitors cocktail. Other conditions as described in Figure 2.

Metabolic products of ^125^I-Ang-(1–12) were analyzed by HPLC in the cultured medium of WKY and SHR cardiac myocytes in the absence and in the presence of inhibitors for ACE, NEP, ACE2, and chymase. In the absence of RAS enzyme inhibitors, ^125^I-Ang-(1-12) was processed into Ang I, Ang II, Ang-(1–7), Ang-(1–5) and other smaller angiotensin peptide fragments in both WKY and SHR (Figure 3). In contrast, the addition of all RAS enzyme inhibitors reduced formation of angiotensin peptides and revealed a larger peak of ^125^I-Ang-(1–12) in the medium of both strains. These data demonstrate the existence of Ang-(1–12) convertases in the medium collected from neonatal cardiac myocytes.

### Contributions of Specific RAS Enzymes in ^125^I-Ang-(1–12) Metabolism

Table 1 summarizes the effects of selective inhibition of ACE, NEP, ACE2, and chymase on ^125^I-Ang-(1–12) metabolism in the culture medium from both WKY and SHR. When the study was performed in the absence of RAS inhibitors, a significantly larger percentile of ^125^I-Ang-(1–12) was rapidly metabolized into Ang fragments [Ang I, Ang II, Ang-(1–7), Ang-(1–4) & smaller peptides] in the medium by WKY and SHR myocytes [only 26% and 8% of ^125^I-Ang-(1–12) remained un-metabolized in the medium, respectively]. However, in the presence of all RAS inhibitors, 90% and 71% of ^125^I-Ang-(1–12) remained in the medium of WKY and SHR cardiac myocytes, respectively. Table 1 also shows that SHR medium from cultured myocytes degraded ^125^I-Ang-(1–12) at a rate faster than the medium from WKY. Sixty minute after addition of the labeled peptide the percentage of ^125^I-Ang-(1–12) detected in the SHR medium is 3-fold less than in WKY; this is associated with lower % fractions of Ang II and significantly higher % fractions of Ang-(1–4) and smaller peptide fragments. In addition, the differences in the percent of ^125^I-Ang-(1–12) and Ang II remaining at the end of the incubation period between WKY and SHR was not affected by the presence of the inhibitor cocktail (Table 1).

**Table 1:** Comparative effects of selective enzyme inhibition on ^125^I-Ang-(1–12) metabolism in medium of cultured neonatal wky and shr cardiac myocytes.

Peptides	No RAS Inhibitors	+All RAS Inhibitors	Minus Lisinopril	Minus SCH39370	Minus MLN-4760	Minus Chymostatin
*Wistar Kyoto Rats*						
Ang-(1–12)*	26±3^a^	90±2	41±7^b^	58±4^b^	92±2	87±3
Ang I	14±1^a^	4±1	18±5^b^	7±2	3±1	5±2
Ang II	37±5^a^	3±1	33±4^b^	6±3	2±1	3±1
Ang-(1–5)	3±1	1±1	2±1	2±1	1±1	1±1
Ang-(1–7)	7±2	1±1	4±1	7±1^b^	1±1	1±1
Ang-(1–4) & smaller peptides	13±3^a^	1±1	2±1	20±2^b^	1±1	3±1
*Spontaneous Hypertensive Rats*						
Ang-(1–12)*	8±2a,c	71±2^c^	27±4^b^	38±4b,c	84±6	51±5b,c
Ang I	10±3	4±1	18±2^b^	8±1	5±2	20±3b,c
Ang II	29±5^a^	14±2^c^	42±4^b^	7±2	6±3	21±4^c^
Ang-(1–5)	6±1	1±1	3±1	1±1	2±1	2±1
Ang-(1–7)	10±2	4±2	3±1	13±2^b^	2±1	2±1
Ang-(1–4) & smaller peptides	37±5a,c	6±2	7±3	33±7^b^	3±1	4±1

Values are expressed as % (Mean ± SE) of Ang-(1–12) unmetabolized* and Ang peptides (%) generated from ^125^I-Ang-(1–12) in the cultured medium incubated for 60 min at 37°C to WKY or SHR myocytes with or without the presence of RAS inhibitors. *No inhibitors group*: Only aminopeptidases inhibitors (amastatin & bestatin) and carboxypeptidases inhibitor (benzyl succinate); *All inhibitors group*: Aminopeptidases and carboxypeptidases inhibitors + RAS inhibitors (lisinopril, SCH39370, MLN-4760 & chymostatin); *Minus RAS inhibitor groups*: One of the RAS inhibitor (lisinopril/SCH39370/MLN-4760/chymostatin) omitted at a time from *all inhibitors group*. All values are expressed as (%) and are the average of three or more independent assays.

*Percent of ^125^IAng-(1–12) parent control remained un-metabolized in the medium exposed to WKY or SHR myocytes for 60 min at 37°C.

a Significantly different (P<0.05) in *No RAS inhibitors* group vs. corresponding WKY or SHR group of +*All RAS inhibitors*.

b Significantly different (P<0.05) vs. corresponding group of +*All RAS inhibitors*.

c Significantly different (P<0.05) vs. corresponding WKY.

In the absence of ACE or the NEP inhibitor, only 41% and 58% of the un-metabolized form of ^125^I-Ang-(1–12) remained in the medium collected from WKY cultured myocytes, respectively, while lower percentages of ^125^I-Ang-(1–12) were found in the medium collected from SHR (Table 1). Removal of lisinopril from the cultured myocytes medium resulted in similar generations of Ang I and Ang II in both WKY and SHR. Absence of either chymostatin or the ACE2 inhibitor (MLN-4760) did not substantially changed the presence of ^125^I-Ang-(1–12) in the medium from WKY cardiac myocytes.

Although, the absence of the ACE2 inhibitor did not change the ^125^I-Ang-(1–12) level in the medium of the SHR myocytes, removal of the chymostatin inhibitor increased production of Ang I (20%) and Ang II (21%). ^125^I-Ang-(1–12) metabolism by SHR myocytes was significantly increased in the absence of chymostatin.

**Figure 4:**
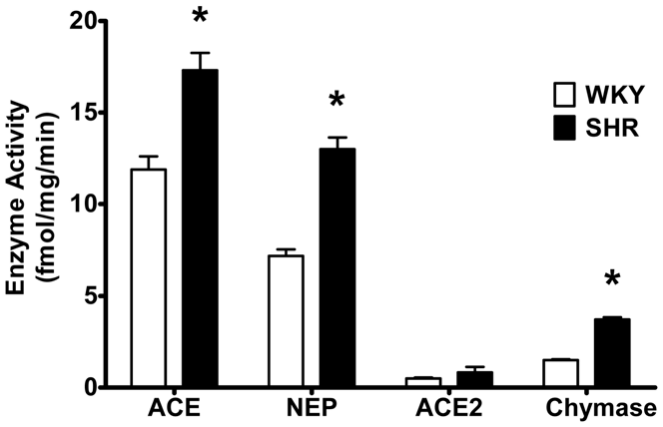
Enzyme activities in WKY and SHR cardiac myocytes. Relative contributions of each enzyme to the ^125^I-Ang-(1–12) metabolism by WKY and SHR cardiac myocytes documents higher enzymatic activity in SHR. These enzymes activity were calculated based on peptide fractions generated from Ang-(1–12) under various RAS inhibitors condition used (see Material and Methods). Abbreviations are: ACE, angiotensin converting enzyme; NEP, neprilysin, ACE2, angiotensin converting enzyme 2. Values are means ± SE. *, p<0.05.

Based on fraction conversions (see Methods), Figure 4 shows the relative contributions of each enzyme to ^125^I-Ang-(1–12) metabolism by WKY and SHR cardiac myocytes. The contribution of ACE, NEP, or chymase enzyme activity to the hydrolysis of ^125^I-Ang-(1–12) was significantly higher in SHR (ACE = 17.3±1.0 fmol·mg protein^−1^·min^−1^; neprilysin = 13.0±0.7 fmol·mg protein^−1^·min^−1^; chymase = 3.7±0.1 fmol·mg protein^−1^·min^−1^, all P<0.05 vs. corresponding WKY) as compared to WKY (ACE = 11.9±0.7 fmol·mg protein^−1^·min^−1^; neprilysin = 7.2±0.4 fmol·mg protein^−1^·min^−1^; chymase = 1.5±0.1 fmol·mg protein^−1^·min^−1^). No significant difference in ACE2 enzyme activity was noted between SHR and WKY (0.83±0.3 fmol·mg protein^−1^·min^−1^ and 0.5±0.1 fmol·mg protein^−1^·min^−1^, respectively).

### Endogenous Localization of Ang-(1–12) in Cardiac Myocytes

**Figure 5:**
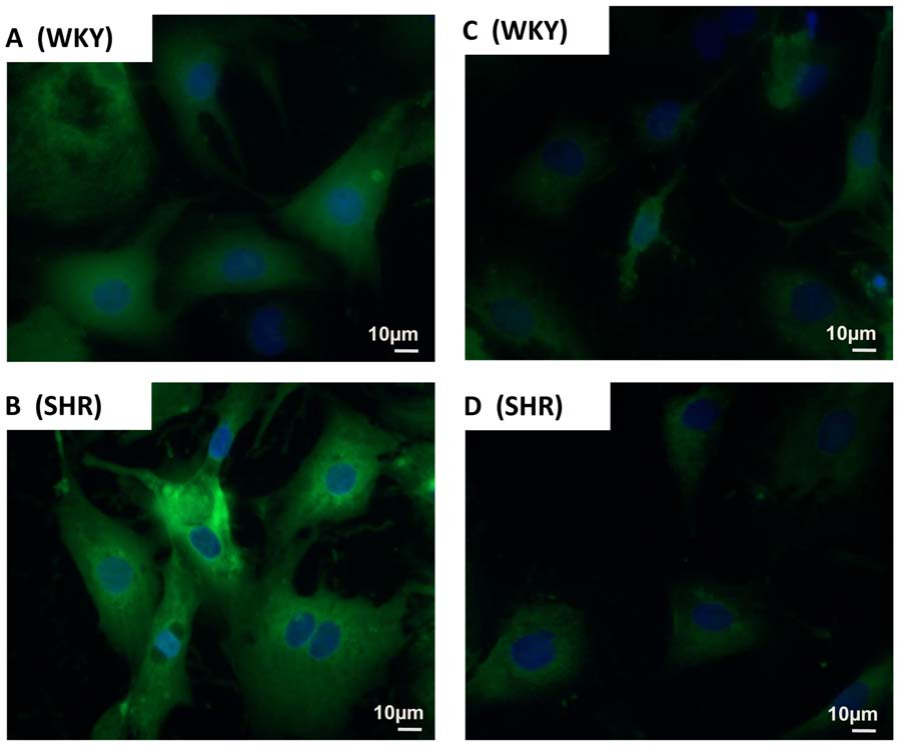
Characteristics of Ang-(1–12) expression in cultured myocytes. Representative examples of fluorescent photomicrographs of Ang-(1–12) immunoreactivity of the cultured myocytes (maintained for 48 hours in serum-deprived medium) from WKY (A and C; *top panel*) and SHR (B and D; *bottom panel*) with protein A purified Ang-(1–12) polyclonal antibody (1∶100 dilution). (A and B) represents the fluorescent staining of endogenous Ang-(1–12) with antibody while (C and D) illustrates the blocking of fluorescent staining of cardiac myocytes after preadsorption of the antibody with 10 µM of synthetic rat Ang-(1–12) peptide. *Magnification, X400.*

The endogenous presence of Ang-(1–12) was visualized by fluorescent staining within the cultured cardiac myocytes of both WKY (top panel) and SHR (bottom panel) in three or more specimens obtained from separate preparations of neonatal cardiac myocytes. As shown in Figure 5, the immunoreactive fluorescent green staining is expressed in the cytoplasm of cardiac myocytes of both WKY (Figure 5 A) and SHR (Figure 5 B). Pre-adsorption of the antibody with 10 µM of synthetic rat Ang-(1–12) peptide blocked the immunoreactive fluorescent green staining of endogenous Ang-(1–12) content in WKY (Figure 5 C) and markedly reduced the immunofluorescence in the myocytes from SHR (Figure 5 D). No green fluorescent staining (only blue staining of nuclei) were found in both WKY and SHR myocytes independently treated with normal Donkey serum in the absence of Ang-(1–12) primary antibody (served as negative controls) (data not shown).

### Angiotensinogen and Renin Expression in Cardiac Myocytes

**Figure 6:**
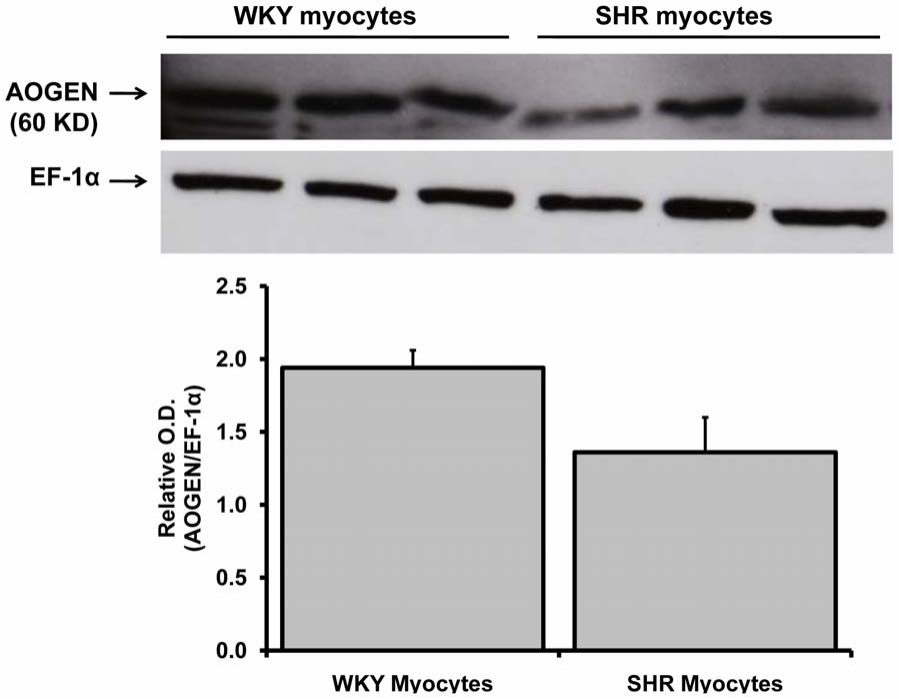
Angiotensinogen protein expression in WKY and SHR myocytes. Western blotting of angiotensinogen (AOGEN) protein expression in neonatal WKY and SHR myocytes (maintained for 48 hours in serum-deprived medium) by using an antibody directed against an epitope on the NH2-terminus region of the angiotensinogen protein (residues 44–56). The myocytes cell lysate (50 µg protein) were separated by gel electrophoresis and transferred on PVDF. Equal protein loading was confirmed by EF-1α detection in cell lysates. Relative O.D. (AOGEN/EF-1 α) is represented as the mean ± SE of three samples from each group.

Angiotensinogen protein expression in the neonatal WKY and SHR cardiac myocytes maintained in serum-free medium for 48 hours was determined by Western blot analysis using appropriate antibodies. The angiotensinogen protein (60 KD band size) was present in both WKY and SHR myocytes (Figure 6). After correcting the angiotensinogen protein expression level with loading control (EF-1α), we observed a tendency for a decreased level of angiotensinogen protein in 48 hours serum deprived neonatal cultured SHR myocytes as compared to WKY. Renin expression in these samples was also analyzed by using a well-characterized renin antibody obtained from Dr. Tadashi Inagami (Nashville, TN). No active renin protein band (37–40 KD) was detected in both WKY and SHR myocytes. These findings further suggest that Ang-(1–12) generated in myocytes from angiotensinogen by a non-renin dependent pathways.

## Discussion

Prior experimental [1], [3]–[8] and pilot studies in human atrial tissue [9] demonstrated that Ang-(1–12) may serve as an alternate pathway for the generation of angiotensin peptides, a pathway that may be of relevance in situations of suppressed renin activity or secretion, as well as possibly acting as an intracellular precursor for the formation of angiotensin peptides. In this study we report for the first time the presence of Ang-(1–12) in neonatal cardiac myocytes from both WKY and SHR together with a time dependent intracellular incorporation of labeled Ang-(1–12) in both strains. Intact uptake of ^125^I-Ang-(1–12) was significantly higher in SHR, a finding that agrees with the previous demonstration of increased Ang-(1–12) content in the myocardium of adult hypertensive rats by both immunohistochemistry and RIA. [3] Assessment of the hydrolysis of ^125^I-Ang-(1–12) in the presence and absence of inhibitors for ACE, NEP, ACE2, and chymase revealed a primary involvement of ACE and NEP in WKY and an additional important contribution of chymase in SHR. This finding is in keeping with reports showing augmented chymase expression and increased chymostatin-inhibitable angiotensin-converting activity in SHR. [10] The increased hydrolytic effects of chymase on ^125^I-Ang-(1–12) metabolism in SHR was associated with a greater processing activity of ACE and NEP. These findings agree with previous studies suggesting increased expression and activity of the cardiac renin angiotensin system in SHR. [10]

Although further work will be necessary to elucidate potential differences in the processing and incorporation of extracellular Ang-(1–12) between neonatal and adult cardiac myocytes previous work shows differences in cellular replication and functional maturation of SHR ventricular myocytes compared to WKY rats during the first postnatal week. [11] This is associated with the presence of earlier development of binucleate myocytes and initiation of hypertrophic myocyte growth in SHR. [12]

The use of a radiolabeled form of Ang-(1–12) allowed for a precise and sensitive quantification of the products generated by the hydrolysis of Ang-(1–12). The drugs were given in doses sufficient to block ^125^I-Ang-(1–12) metabolism by at least 90%. As documented elsewhere, [13]–[15] inclusion of amastatin, bestatin, benzyl succinate, and PCMB in all processed samples was an added precaution to prevent the further degradation of angiotensin peptides by aminopeptidases and carboxypeptidases into smaller fragments.

The heart is an organ where local formation of Ang II is implicated to regulate cardiac remodeling due to increased afterload or ischemia. [16], [17] Central to this concept is the demonstration that Ang II can be produced locally and function as a paracrine [16], [17] or autocrine factor. [18] Through the use of radiolabeled ^125^I-Ang I and ^125^I-Ang II van Kats et al. [19] concluded that most of the Ang I and Ang II found in the heart was produced locally. These findings are reinforced by the recent demonstration of increased Ang II synthesis in cardiac myocytes from diabetic rats. [20] In the SHR, increased activity of the RAS is found in the heart [21], [22] and the kidneys [23] while in cultured newborn SHR heart cells, increased uptake of ^3^H-proline by Ang I and Ang II was abolished by blockade with either an ACE inhibitor or an AT_1_ receptor antagonist. [24] The latter findings suggest an intrinsic increase in protein uptake by SHR myocytes, a finding that is in keeping with the current observations of differences in the rate of ^125^I-Ang-(1–12) incorporation in WKY and SHR myocytes.

A further insight into the functional significance of local RAS is expanded by our finding of an increased incorporation of intact ^125^I-Ang-(1–12) in cardiac myocytes from cultured neonatal cells of SHR. The HPLC analysis of cell lysate showed that intact Ang-(1–12) is internalized by cardiac myocytes, although the amount internalized represents a small fraction of total loaded ^125^I-Ang-(1–12) (<5.2% in SHR & <3.6% in WKY). Further, we report around 50% inhibition of cellular internalization of radiolabeled Ang-(1–12) in the presence of excess cold Ang-(1–12) [Iodine-unlabeled Ang-(1–12); 10^3^ µM]. We suggest that the failure to achieve higher inhibition may be due to differences in binding affinity between cold Ang-(1–12) and ^125^I-Ang-(1–12) or the potential existence of several uptake mechanisms. This interpretation is in keeping with the finding that no more than only 50% of bound angiotensinogen could be displaced from human renal tubular epithelial cells [25]. As in their study, Ang-(1–12) as an extended form of Ang I, might bind to a low affinity receptor or alternatively to the same angiotensinogen receptor characterized by Yayama et al., [26] and Pan et al. [25]. In addition, Mineo et al., [27] reported a similar 50% inhibition of ^125^I-Ang II in the presence of unlabeled Ang II in endothelial cell monolayers. They interpreted these findings as suggesting that about 50% of Ang II was transported via an intracellular pathway while the remainder was accounted for non-specific transport. The augmented incorporation of Ang-(1–12) into SHR myocytes agrees with the reported observation of increased incorporation of ^3^H-uradine and ^14^C-leucine into 10-day-old SHR heart cells. [28] The process appears to remain present in older animals as adult SHR showed increased Ang-(1–12) expression as determined independently by immunostaining of left ventricular sections with a selective Ang-(1–12) antibody and additional measures of Ang-(1–12) content by radioimmunoassay. [3]

In the controlled environment of a cell culture system, neonatal cardiac myocytes of SHR and WKY metabolized ^125^I-Ang-(1–12) into Ang I, leading to sequential production of Ang II, Ang-(1–7), and other smaller angiotensin fragments. Ang-(1–12) metabolism by cardiac myocytes was significantly blocked in the presence of specific enzyme inhibitors for ACE, neprilysin, ACE2, and chymase. While the hydrolysis of ^125^I-Ang-(1–12) was found to be primarily due to ACE and NEP in WKY rats, chymase contributed to the conversion of ^125^I-Ang-(1–12) to Ang I and Ang II in SHR. This new observation is in keeping with the demonstration of an important role for mast cell derived chymase in Ang II formation following either decreased expression [29] or pharmacological blockade of ACE [30] This finding also agrees with Prosser et al. [29] report that chymostatin completely inhibited Ang II formation from Ang-(1–12). As discussed elsewhere, [5], [31] differences in the nature of the enzymatic pathways accounting for Ang-(1–12) metabolism in the studies reported by Nagata et al. [1] and Prosser et al. [8] may be a result of increased cellular permeability associated with the isolated cardiac perfusion procedures use by Prosser et al. (12). The more controlled preparation employing only cardiac myocytes cultures rather than the whole organ or the systemic circulation provided a more precise system for the identification of the differential routes of Ang-(1–12) processing reported in this study. In addition, the higher contribution of chymase in Ang-(1–12) metabolism found in SHR compared to WKY controls is in keeping with previous findings of increased chymase activity in SHR. [32]

Immunofluorescence staining using a well-characterized Ang-(1–12) antibody clearly shows the presence of Ang-(1–12) in neonatal cultured SHR and WKY myocytes. In addition, angiotensinogen protein but not renin was also detected in these myocytes by Western blot analysis. These studies further buttress the hypothesis that Ang-(1–12) may be generated intracellularly from angiotensinogen as well as incorporated into the myocyte.

In summary, we document the incorporation of Ang-(1–12) and metabolic pathways of Ang-(1–12) in cardiac myocytes of WKY and SHR. Increase cellular uptake of Ang-(1–12) in the cardiac myocytes from SHR is in keeping with our previous demonstration of elevated values of Ang-(1–12) in the myocardium of SHR. [3] Since Ang-(1–12) processing was demonstrated by us to occur both *in vivo* [5] and *in vitro* [4] via a non-renin pathway, the current identification of ACE, NEP, and chymase reveals the enzymatic pathways contributing to the generation of angiotensin peptides from Ang-(1–12) in the medium collected from neonatal myocytes.

## Materials and Methods

### Isolation of Neonatal Cardiac Myocytes

15–18 days pregnant SHR and normotensive WKY mothers were purchased from Charles River Laboratories Inc. (Wilmington, MA). Neonatal rat ventricular cardiac myocytes of WKY and SHR pups (1–3 day old) were isolated by proteolytic digestion and differential plating, as previously described. [33], [34] Briefly, neonatal pups were placed under deep isoflurane (4%) anesthesia and their hearts were quickly removed and placed into ice cold Hanks Balanced Salt Solution (HBSS) medium. The myocytes were isolated from minced ventricles by proteolytic digestion and differential plating (to separate the myocytes from fibroblast). Myocytes were collected and maintained in DMEM/F-12 supplemented with 10% FBS, 10 µg/mL insulin, 10 µg/mL holo-transferrin, 100 µM 5-bromodeoxyuridine (to prevent proliferation of non-myocytes), and antibiotics (ampicillin and streptomycin) in a humidified incubator with an atmosphere of 95% O_2_, 5%CO_2_. Myocytes were incubated overnight in the same DMEM/F-12 medium in the absence of FBS and 5-bromodeoxyuridine before use for ^125^I-Ang-(1–12) uptake and metabolism studies. Cells isolated by this protocol routinely showed positive staining for anti-sarcomeric myosin (1∶100 dilutions, Sigma-Aldrich Co., St Louis, MO) and no immunoreactivity labeling with antibodies to alpha-actin, fibronectin, or vimentin (1∶100 dilutions, Sigma-Aldrich Co., St Louis, MO).

### Ethics Statement

All experimental procedures (Animal Protocol # A09-026 approved on March 11, 2009 by Dr. Mark S. Miller, Chairman of Animal Care and Use Committee) were performed in accordance with guidelines set forth by the Institutional Animal Care and Use Committee of the Wake Forest University School of Medicine.

### Cellular Uptake and Metabolism of ^125^I-Ang-(1–12) Assays

Cellular uptake and metabolism of Ang-(1–12) by WKY and SHR cultured myocytes were studied under different combinations of RAS inhibitors. To elaborate, lisinopril for ACE, MLN-4760 for angiotensin-converting enzyme 2 (ACE2), SCH39370 for neprilysin (NEP) and chymostatin for chymase as detailed in Table 2.

**Table 2:** Outline of enzyme inhibitors employed in the experiments.

Group	Inhibitors added (10 µM each)
*No RAS inhibitor group*	Only peptidases inhibitors (amastatin, bestatin & benzyl succinate) and P-chloromercuribenzoate (PCMB).
*All RAS inhibitor group*	Above inhibitors + RAS inhibitors [lisinopril for ACE, MLN-4760 for ACE2, SCH39370 for NEP and chymostatin for chymase].
*Minus RAS inhibitor groups*	All above inhibitors except one of the RAS inhibitor (lisinopril, SCH39370, MLN-4760 or chymostatin) was omitted at a time from the reaction mixture.

Abbreviations as in text.

In cellular uptake studies, twenty-four hour serum deprived WKY or SHR myocytes, washed twice with serum-free DMEM/F-12 medium, were exposed to culture medium containing ^125^I-Ang-(1–12) (1 nmol/L) at specific activity 3,900 cpm/fmol in the presence of all RAS inhibitors each added at a concentration of 10 µM at 37°C in a water bath for 0–30 min. Inhibitors were added 15 min prior to adding the ^125^I-Ang-(1–12). To determine the specificity of cellular uptake of radiolabeled ^125^I-Ang-(1–12), the cultured cardiac myocytes in some of the experiments, were co exposed to excess amount of unlabeled Ang-(1–12) [1 µmol/L] along with ^125^I-Ang-(1–12) for 30 min at 37°C. Afterward, the dosing medium was removed and the cells were washed three times with ice-cold PBS and one time with 0.05 M glycine-HCl (pH 4.0) to remove the membrane bound ^125^I-Ang peptides. After removing the membrane bound ^125^I-Ang peptides, the cells were lysed with 1 N NaOH and counted in a gamma counter to determine the total cellular uptake of ^125^I-Ang-(1–12). In other experiments WKY or SHR cardiac myocytes were also exposed to ^125^I-Ang-(1–12) in the absence and presence of RAS inhibitors for 60 min at 37°C as described above. At the end of exposure time, the washed cells were collected in 1 mL of acid-ethanol (0.1N HCl+80% ethanol) and stored at −80°C to analyze the cellular uptake of ^125^I-Ang-(1–12) by HPLC. Total cellular protein concentration were measured by Bradford Reagent using bovine serum albumin (BSA) as standard and the total cellular counts were normalized in terms of mg protein.

For ^125^I-Ang-(1–12) metabolism study, the cultured myocytes (WKY or SHR) were preincubated for 15 min under various combinations of RAS and peptidases inhibitors (10 µmol/L of each) as described above. After preincubation of myocytes with the inhibitors, ^125^I-Ang-(1–12) (1 nmol/L) was added to reaction medium and incubated for 60 min at 37°C in the water bath. At the end of incubation time, exposed medium and cardiac myocytes were collected separately and processed for HPLC analysis as described below.

### HPLC Analysis of Ang-(1–12) Metabolic Products in Medium and in Cardiac Myocytes

Cellular uptake of ^125^I-Ang-(1–12) by cardiac myocytes and their metabolic products in cell lysate and dosing medium were analyzed by HPLC as previously described. [35], [36] The medium from the cell cultures was removed and mixed with 1% of phosphoric acid and stored at −80°C until processing the samples for Ang contents [Ang-(1–12), Ang I, Ang II and Ang-(1–7)] by HPLC. After removing the dosing medium, myocytes were washed three times with ice-cold PBS and one time with 0.05 M glycine-HCl (pH 4) to remove the membrane bound ^125^I-Ang. After removing the membrane bound ^125^I-Ang-(1–12), the myocytes were scraped using a cell lifter in 1 mL of acid-ethanol (0.1N HCl + 80% ethanol). The scraped cells were immediately frozen and stored at −80°C till processing the samples to detect the cellular ^125^I-Ang content by HPLC.

Before using the samples for HPLC analysis, the cell lysate and medium were passed through Sep-Pak C18 cartridge columns (Waters Corp., Milford, MA) to collect the ^125^I-labeled Ang-(1–12) metabolic products. Briefly, cells and medium (stored at −80°C) were thawed in ice and the cell lysate were briefly sonicated for 10 sec. The lysed cells were centrifuged at 28,000 rpm for 15 min to remove the cell membrane and debris. The clear supernatant and the medium were diluted with 9x vol. of 0.1% trifluoroacetic acid (TFA) and were passed through activated Sep-Paks by gravity. The columns were first washed with 10 mL of 0.1% TFA and then with 5 mL of MilliQ water. After washing the column, the bound ^125^I-labeled Ang-(1–12) metabolic products were eluted with 80% methanol + 0.1% TFA. Finally, Sep-Pak eluted samples were analyzed by HPLC to detect the ^125^I-Ang-(1–12) metabolic products. We used a linear gradient from 10% to 50% mobile phase B at a flow rate of 0.35 mL/min at ambient temperature. The solvent system consisted of 0.1% phosphoric acid (mobile phase A) and 80% acetonitriles/0.1% phosphoric acid (mobile phase B). The eluted ^125^I products were monitored by an in-line flow-through gamma detector (BioScan). Products were identified by comparison of retention times to synthetic [^125^I] standard peptides and the data were analyzed with Shimadzu (version 7.2.1) acquisition software. The iodination of rat Ang-(1–12) and other angiotensins was performed as described previously. [35], [36]

### Contribution of specific enzymes (ACE, NEP, ACE or chymase)

The contribution of ACE, NEP, ACE2 or chymase to the hydrolysis of ^125^I-Ang-(1–12) substrate were analyzed by measuring the amount of Ang products formation in the cultured medium after exposing the cells in the presence of all RAS inhibitors cocktail and in the absence of specific enzyme inhibitors for ACE, NEP, ACE2 or chymase only (as described above). Medium was collected after exposing the WKY and SHR cells for 60 min at 37°C under two different enzyme inhibitors conditions (+All RAS inhibitors *versus* minus ACE/NEP/ACE2/chymase inhibitor only) and were analyzed by HPLC as described above. The enzyme activity was calculated based on amount of 1 nM of ^125^I-Ang-(1–12) substrate added to the reaction mixture and metabolized into final products by specific RAS enzyme. The protein content of each well was determined by Bradford Reagent using BSA as standard protein. Experiments were performed three or more times and the enzyme activity values were reported as fmoles of Ang product formation from ^125^I-Ang-(1–12) substrate per min per mg protein (fmol·mg protein^−1^·min^−1^).

### Localization of Endogenous Synthesis of Ang-(1–12) in Cultured Cardiac Myocytes

The presence of Ang-(1–12) in the cultured cardiac myocytes were evaluated by immunofluorescent microscopy using a protein A column purified anti-rabbit rat Ang-(1–12) primary antibody (1∶100 dilution). We showed previously that the Ang-(1–12) antibody did not cross-react with angiotensinogen, Ang I, Ang II and Ang-(1–7) and the fluorescence Cy2 secondary antibody (Jackson ImmunoResearch Laboratory, Inc., West Grove, PA). [3] Cardiac myocytes from WKY and SHR were cultured as described above in 8-well chambered slides. Myocytes (maintained in serum-deprived medium for 48 hours) were then washed three times with PBS, fixed in 4% paraformaldehyde for 15 min and permeabilized in 0.1% Triton X-100 in 4% paraformaldehyde for another 15 min. The fixed cells were incubated overnight at 4°C with the purified rat Ang-(1–12) antibody (1∶100 dilutions). The next day, the cells were washed three times with PBS and incubated with the fluorescence tagged secondary antibody (Cy^Tm^2-conjugated AffiniPure Donkey Anti-Rabbit IgG; 1∶200 dilutions) for 1 h at room temperature. The secondary antibody was washed three times with PBS and cells were covered with cover slip after adding one drop of ProLong Gold antifade reagents with DAPI (4′–6-diamidino-2-phenylindole; a blue fluorescent probe widely use to stain DNA in the nuclei of cells). The resultant fluorescent immunoreactive staining of endogenous rat Ang-(1–12) in cardiac myocytes of WKY and SHR cells were visualized using a fluorescent microscope (Leica DM 4000B, Leica Microsystems, Wetzlar, Germany). The immunofluorescent staining was repeated three or more times in cultured cardiac myocytes isolated from different batches of WKY and SHR neonatal pups.

### Western blot analysis of Angiotensinogen and Renin in Cultured Cardiac Myocytes

The expression of angiotensinogen protein in neonatal cultured cardiac myocytes was assessed by immunoblot. Myocytes were isolated and maintained as describe above. Before collecting the myocytes for angiotensinogen protein expression, the cells were cultured for 48 hours in serum-deprived medium. Angiotensinogen protein expression was assessed in total cell lysate by using an antibody directed against an epitope on the NH_2_-terminus region of the protein (residues 44–56) which detects both Ang I-containing and des-Ang I forms of the protein. [37] The myocytes cell lysate (50 µg protein) were separated by gel electrophoresis and transferred to polyvinylidene difluoride membranes (PVDF). The PVDF membranes were probed with an antibody against rat angiotensinogen protein (1∶1000 dilutions). Densities of the 60-KD immunoreactive bands of WKY and SHR groups were determined with a MCID imaging system (MCID Elite 7.0, Imaging Research Inc., St. Catharine, ON, Canada). The expression of renin in neonatal cultured myocytes was also assessed using an antibody against rat renin (1∶1000 dilution) obtained from Dr. Tadashi Inagami as described above.

### Reagents

Angiotensin-(1–12) (>99% purity) was purchased from GenScript USA Inc. (Piscataway, NJ). DMEM/F12, Hanks' Balance Salt Solution (HBSS), Leibovitz's L-15 medium, fetal bovine serum (FBS), penicillin and streptomycin were purchased from GIBCO Invitrogen (Gaithersburg, MD). ProLong Gold antifade reagent with DAPI was purchased from Invitrogen (Molecular Probes, Inc., Eugene, OR). Fluorescent secondary antibody (Cy^Tm^ 2-conjugated AffiniPure Donkey Anti-Rabbit IgG) and normal Donkey serum were purchased from Jackson ImmunoResearch Laboratories, Inc. (West Grove, PA). Collagenase, trypsin and soybean trypsin inhibitors were purchased from Worthington Biochemical Corporation (Lakewood, NJ). Lisinopril (ACE inhibitor) and SCH39370 (neprilysin inhibitor) were obtained from Merck (West Point, PA). MLN-4760 (ACE2 inhibitor) was obtained from Millennium Pharmaceuticals (Cambridge, MA). Chymostatin (chymase inhibitor), amastatin, bestatin and benzyl succinate and PCMB were purchased from Sigma-Aldrich Co. (St. Louis, MO). Radioactive ^125^I was purchased from PerkinElmer Life and Analytical Sciences, Inc. (Waltham, Massachusetts). All other chemicals used in this study were of analytical grade and were obtained from Sigma (St. Louis, MO), and Fisher Scientific (Atlanta, GA).

### Statistical Analysis

Experiments were repeated independently three or more times. All values are reported as mean ± SE. The Student's *t*-test and repeated-measures ANOVA followed by a Turkey's post hoc test for multiple comparisons were used to determine significant differences at a probability of 0.05 using GraphPad Prism 5.0 software (San Diego, CA).
